# Antimicrobial Activity Versus Virulence Potential of Hyaluronic Acid: Balancing Advantages and Disadvantages

**DOI:** 10.3390/ijms262311549

**Published:** 2025-11-28

**Authors:** Kamila Korzekwa, Kamil Sobolewski, Miriam Wiciejowska, Daria Augustyniak

**Affiliations:** 1 Department of Microbiology, Faculty of Biological Sciences, University of Wroclaw, Przybyszewskiego 63, 51-148 Wroclaw, Poland; 2 Sobolewscy Medical Academy, Grabiszyńska 252a, 53-234 Wrocław, Poland; 3 Department of Pathogen Biology and Immunology, Faculty of Biological Sciences, University of Wroclaw, Przybyszewskiego 63, 51-148 Wroclaw, Poland; daria.augustyniak@uwr.edu.pl

**Keywords:** hyaluronic acid, antimicrobial activity, virulence, biofilm, hyaluronidase, immunomodulation, therapeutic potential

## Abstract

Hyaluronic acid (HA) is a ubiquitous glycosaminoglycan essential for maintaining tissue hydration, structural integrity, and immunological homeostasis in vertebrates. Although traditionally regarded as a host-derived molecule, HA is also produced by a range of microorganisms, most notably *Streptococcus* spp., through specialized hyaluronan synthases (HAS). Microbial HA and host-derived HA fragments play key roles not only in tissue physiology but also in infection biology, influencing microbial virulence, biofilm formation, and immune evasion. In bacteria, HA-rich capsules promote adhesion, shield pathogens from complement-mediated opsonization and phagocytosis, and facilitate dissemination through host tissues. Conversely, HA-degrading enzymes and reactive oxygen species generate low-molecular-weight HA fragments that amplify inflammation by activating—toll-like receptor 2 (TLR2)/toll-like receptor 4 (TLR4) signaling, contributing to chronic inflammatory states. Furthermore, microbial HA modulates biofilm organization in both bacterial and fungal pathogens, enhancing persistence and antimicrobial tolerance. Clinically, widespread use of HA-based dermal fillers has generated increasing concern over delayed biofilm-associated infections, diagnostic challenges, and complications arising from microbial contamination and host–microbe interactions. Recent advances in HA engineering, including anti-microbial HA conjugates and receptor-targeted biomaterials, offer promising strategies to mitigate infection risk while expanding therapeutic applications. This review synthesizes current knowledge on HA biosynthesis across biological kingdoms, its dualistic role in health and disease, and its emerging relevance at the interface of microbiology, immunology, and biomedical applications.

## 1. Introduction

Hyaluronic acid is widely distributed in nature across two domains of life—Eukarya and Bacteria. In living organisms, it takes the form of its sodium salt, sodium hyaluronate, which is more accurately referred to as hyaluronan. It has been found in several biological kingdoms, including animals (e.g., humans; mammals like rabbits; cattle; birds like roosters; and mollusks [1]; protists (e.g., *Chlorella* sp. algae infected with chlorovirus) [2,3]; fungi (restricted to yeasts *Cryptococcus neoformans*) [2]; and procaryotes, including bacteria such as *Streptococcus equi*, *Streptococcus zooepidermicus*, *Streptococcus equisimilis*, *Streptococcus pyogenes*, *Streptococcus uberis*, and *Pasteurella multocida* [2,4,5]. Conversely, its presence in other fungi, plants, and insects has not yet been confirmed [6].

In 1961, HA was used for the first time in medicine during surgery on a damaged retina, and in 1980, trials began on its application in esthetic medicine. In Europe, HA was first employed as a wrinkle filler in 1980 [7]. Initially, HA was isolated from bovine eyes and obtained from animal sources, such as rooster combs. Advances in biotechnology and genetic engineering have made it possible to obtain HA under laboratory conditions using recombinant bacteria specifically designed for this purpose [8].

The findings of clinical and translational studies indicate that hyaluronan (HA) has the capacity to impede the process of pathogen adhesion, hinder the formation of biofilms, reduce the expression of virulence-associated determinants, and reinforce the integrity of epithelial barriers and immune system. Furthermore, HA functions as a multifaceted carrier, facilitating targeted antibiotic delivery and potentiating antimicrobial activity. However, these effects must be regarded as highly context-dependent [8].

It is important to note that HA is not merely a passive barrier, but rather an active participant in host–pathogen crosstalk. Consequently, its biological impact reflects a dynamic, bidirectional interaction: bacteria sense and metabolize HA, modulating their virulence programs, while HA simultaneously shapes host immune responses and maintains epithelial integrity. The present article aims to explore both aspects of these interactions, i.e., how HA affects microbial behavior and how microbes, in turn, alter HA signaling and function, thereby providing a balanced view of its antimicrobial promise and potential risks.

## 2. Structure of Hyaluronic Acid

Hyaluronic acid is notably one of the most diverse substances, especially when considering its multitude of properties and applications [9]. Its remarkable functionality has paved the way for numerous advancements across various fields of science [10]. Although its full chemical make-up was discovered in the 1950s, its identification dates back to 1934, when it was first described by John Palmer and Karl Meyer [7,11,12]. Deceptively simple in structure, this glycosaminoglycan, composed of repeating disaccharide chains of N-acetyl-glucosamine and glucuronic acid, constitutes a pivotal component in nearly all biological processes [3,13].

**Figure 1:**
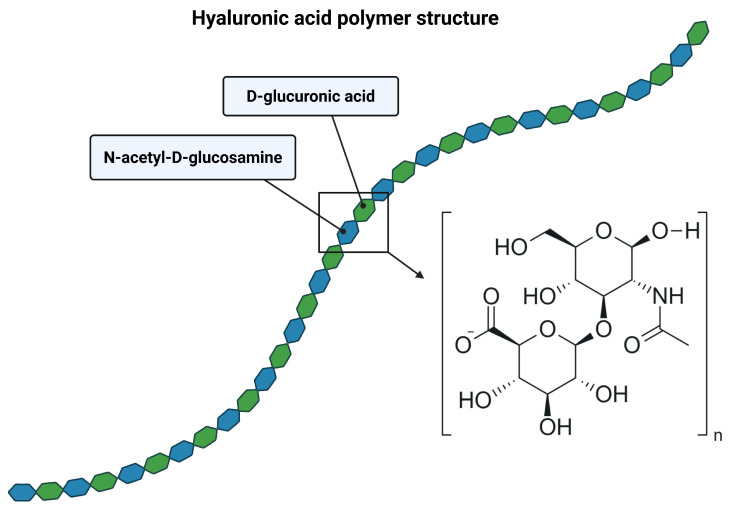
Structure of HA polymer. The structural configuration of D-glucuronic acid (illustrated in green) and N-acetyl-D-glucosamine (illustrated in blue). Each disaccharide unit is linked via alternating β-1,3 and β-1,4 glycosidic bonds, forming a linear, non-sulfated glycosaminoglycan. Created by BioRender.com [19].

HA belongs to the group of glycosaminoglycans (GAGs). It is a linear heteropolysaccharide with a high molecular weight. All glycosaminoglycans (e.g., chondroitin sulfate, dermatan sulfate, keratin sulfate, heparin) are composed of repeating disaccharide residues composed of an amino sugar (N-acetylgalactosamine or N-acetylglucosamine) and a uronic acid (glucuronic acid, iduronic acid, or galactose) [13,14]. HA is composed of repeating disaccharide units linked by a β-(1-4) glycosidic bonds. Each disaccharide is composed of N-acetyl-D-glucosamine and D-glucuronic acid residues linked by a β-(1-3) glycosidic bond (Figure 1). Unlike other GAGs, HA is not sulfated and is not covalently linked to proteins. It is the only natural non-sulfated GAG identified to date [15]. Furthermore, its synthesis occurs on the inner surface of the cell membrane, whereas other GAGs are synthesized via enzymatic pathways in the Golgi apparatus. HA can reach very high molecular weights (as high as 10^8^ Da), whereas other GAGs exhibit significantly lower molecular weights (maximum 5 × 10^4^ Da; typically 1.5–5 × 10^4^ Da) [13,14,15,16].

As mentioned above, the flexibility and solubility of HA polymers result from β-1,3 links, which, together with β-1,4 bonds, connect the saccharide units that form the overall structure [10,13]. The number of polymer units determines the molecular weight of HA, which in turn allows it to be classified into four forms, closely linked to different biological and chemical functions: very low-, low-, medium-, and high-molecular-weight [9,10,17]. For instance, very-low-molecular-weight hyaluronan (below 50 kDa) is often associated with pro-inflammatory responses as well as cytotoxic effects [17]. On the other hand, low-molecular weight hyaluronan (50 to 200 kDa) shows antiapoptotic activity, and promotes cell movement; it also finds application in tissue hydration and healing [10,11,18]. Medium sized HA (750 to 1000 kDa) is linked to embryogenesis, ovulation and wound healing, whereas high-molecular-weight hyaluronan (1000 to 2000 kDa) has antiangiogenic, immunosuppressive, anti-inflammatory properties, inhibiting cell proliferation and movement contrary to the low molecular hyaluronic acid [8,9,17]. Notably, the high-molecular-weight form of HA is the one most commonly used as a medical-grade space filler [10,18].

### Receptors for Hyaluronic Acid

The cellular and molecular mechanisms underlying HA recognition and signaling involve a sophisticated network of receptors and binding proteins that exhibit both conserved and species-specific features across biological kingdoms. The primary HA receptor CD44 exists in multiple splice variants that recognize different HA molecular weight ranges and activate distinct signaling cascades. These include pathways involving calcium-dependent kinases (CaMKKβ), AMP-activated protein kinase (AMPKα), and autophagy regulatory proteins (ULK1). These mechanisms have recently been demonstrated in murine macrophage host cells (RAW 264.7 and bone-marrow-derived macrophages) during HA’s mediated internalization of the fungal pathogen *Cryptococcus gatti* [18,20]. Recent studies conducted in diverse human and murine cell models, as well as in transgenic Alzheimer’s disease mice, further reveal that CD44 engagement triggers complex intracellular trafficking programs that can either promote cell survival or apoptotic responses, depending on HA fragment size and cellular context [16,21,22,23]. Additional HA-binding proteins include; (i) RHAMM (receptor for HA-mediated motility), which mediates HA-dependent cell migration and proliferation through interactions with the cytoskeleton and cell cycle machinery, (ii) LYVE-1 (lymphatic vessel endothelial HA receptor 1) that facilitates HA uptake and degradation in lymphatic tissues, and (iii) TSG-6 (TNF-stimulated gene 6) that functions as both an HA-binding protein and a catalyst for HA cross-linking through its link module and TSG-6 domain architecture [8,24]. Among the additional HA-binding proteins, LYVE-1 has also been examined in infection-related contexts, including viral infection models (e.g., Zika virus) on human trophoblast cell lines. In contrast, the roles of RHAMM and TSG-6 described here originate primarily from mammalian cell physiology studies [24,25].

## 3. Synthesis of Hyaluronic Acid

Hyaluronic acid is a natural biopolymer that shares the same chemical structure in humans, other vertebrates, and bacteria [10,17]. However, its synthesis differs between Eukaryota and Bacteria in terms of the site of synthesis and mode of export. While in higher Eukaryota HA synthesis occurs in the plasma membrane with hyaluronan synthases, followed by extrusion of the HA polymer directly into the extracellular matrix (ECM), in bacteria HA is synthesized by a single membrane-bound enzyme known as hyaluronan synthase (HasA) encoded within the has operon. Produced in this way very high-molecular-weight HA is directly secreted onto the cell surface and this process is tightly linked to capsule formation [9,21].

Fungi rarely harbor native HA biosynthetic pathways; instead, HA production in fungal systems generally relies on heterologous expression of bacterial or mammalian HAS genes supported by engineered precursor pathways. A detailed description of synthesis in vertebrates and microorganisms is presented in the following subsections.

### 3.1. Hyaluronic Acid Synthesis in Vertebrates

In vertebrates HA is synthesized at the inner surface of the cell membrane by different hyaluronan synthases (HAS), a family of three of transmembrane glycosyltransferase isoenzymes known as HAS1, HAS2, and HAS3 (Table 1). Each of these enzymes is composed of two catalytic domains. One domain catalyzes the transglycosylation of D-glucuronic acid, while the second catalyzes the transglycosylation of N-acetyl-D-glucosamine. Hyaluronan synthases alternately attach glucuronic acid and N-acetylglucosamine to the reducing end of the growing chain, forming a linear polymer chain composed of repeating disaccharides. This mechanism enables the biosynthesis of extremely long hyaluronan polymers with molecular weights reaching up to 10^7^ Da. The resulting HA chains are transported to the cell surface or into the ECM through the cell membrane and HAS protein complexes [26,27,28]. This process leads to the formation of a protective coating around the cell, allowing HA to perform antioxidant functions and maintain an environment with an optimal degree of hydration [28,29].

Although all three HAS catalyze the polymerization of HA, they differ in biochemical properties, enzymatic activity and in the size of the HA they produce. HAS1 has relatively low activity, and generates HA of variable length with a molecular weight of 2 × 10^5^ to 2 × 10^6^ Da. HAS2 is an enzyme with higher catalytic activity, that enables the synthesis of chains with a molecular weight greater than 2 × 10^6^ known as high-molecular-weight HA. HAS2 is responsible for hyaluronan synthesis in normal, mature cells [30]. It also plays a role in tissue development and repair. However, it may be involved in inflammation, tumorigenesis, pulmonary fibrosis, and scar tissue formation [30,31,32]. The most active HAS isoenzyme is HAS3, which produces shorter HA molecules up to 3 × 10^5^ Da and functions mainly in rapidly remodeling tissues [33,34]. Dysregulation or abnormal expression of HAS genes can lead to altered HA production, which in turn may contribute to neoplastic transformation and metastasis [29,30,31,35].

Recent studies have shed new light on how HA synthesis is regulated. In naked mole rat HAS2 produces ultra-high-molecular-weight HA with enhanced cancer resistant properties. In Alzheimer’s disease, interactions between amyloid-β precursor protein (AβPP-tau) and HAS1 have been linked to changes in gene activity through nuclear speckle formation and gene transcription regulation. In cancer, ransforming growth factor β (TGF-β)and reticular activating system, (RAS) signaling have been shown to work together to activate “primed” enhancers that drive HAS2 expression, thereby promoting metastasis [23,36,37]. The evolutionary relationships among HAS enzymes suggest ancient origins with subsequent lineage-specific duplications and functional diversification. Phylogenetic analyses reveal that bacterial and eukaryotic HAS enzymes likely evolved from common GT-2 ancestors. However, they have undergone extensive structural modifications to accommodate different cellular architectures, membrane systems, and regulatory requirements, ultimately resulting in the remarkable diversity of HA synthesis strategies observed across the tree of life [24,27].

### 3.2. Degradation of Hyaluronic Acid in Vertebrates

HA degradation in the body can occur via specific enzymes (hyaluronidase—HYAL, β-D-glucuronidase and β-N-acetyl-hexosaminidase), or as a result of oxidation via reactive oxygen species (ROS) [34] HYAL enzymes belong to a group of endoglycosidases that have the property of randomly cleaving the β-N-acetyl-D-glucosamide (1,4-glycosidic bonds) in HA. Six HYAL enzymes have been identified: HYAL1, HYAL2, HYAL3, HYAL4, PH20/SPAM1, and HYAL-P1 [31,33,38].

The best-known are HYAL1, HYAL2, and PH20/SPAM1. Both HYAL1 and HYAL2 are active at acidic pH (pH < 4) and have potent activity in human somatic cells [31,33,39,40,41]. HYAL1 is responsible for the regulation of multiple cellular processes, including apoptosis, and it is a significant contributor to cellular metabolism, which may therefore act as a tumor enhancer and in angiogenesis [30]. HA degradation occurs with the participation of HYAL1 and HYAL2, but the mechanisms are not fully understood. Unlike HYAL1 and HYAL2, PH20/SPAM1 is a multifunctional enzyme. It exhibits endoglycosidase activity in both acidic and neutral environments and is also involved in fertilization-related processes [40].

HA may also be degraded by reactive species, which are associated with inflammation, and may contribute to oncogenesis [33,34,42]. During the breakdown of HA, ROS lead to the cleavage of glycosidic bonds and subsequent depolymerization [43]. Moreover, excessive ROS activity has been reported as a trigger for this degradation pathway [42]. In the joint environment, HA depolymerization reduces the viscosity of synovial fluid, which in turn promotes cartilage damage, pain, and stiffness [44]. Thus, ROS-mediated HA degradation influences the antioxidant capacity of HA and may play a role in disease progression. Further studies are needed to support this hypothesis.

### 3.3. Hyaluronic Acid Synthesis in Microorganisms

Certain microorganisms inherently synthesize hyaluronic acid, whereas others do not produce it naturally but can be engineered through genetic transformation to enable large-scale industrial production. Information on this topic is described in the following subsections of this section.

#### 3.3.1. Hyaluronic Acid Synthesis Among Bacteria

To date HA presence has been confirmed in several bacterial strains, including groups A and C streptococci (e.g., *S. pyogenes* and *S. equi*), *Pasteurella multocida*, *Bacillus anthracis*, and *Haemophilus influenzae* [8,45,46,47]. These microorganisms have a natural ability to produce and secrete HA as a secondary metabolite, which is incorporated into virulence-associated capsules [10].

**Figure 2:**
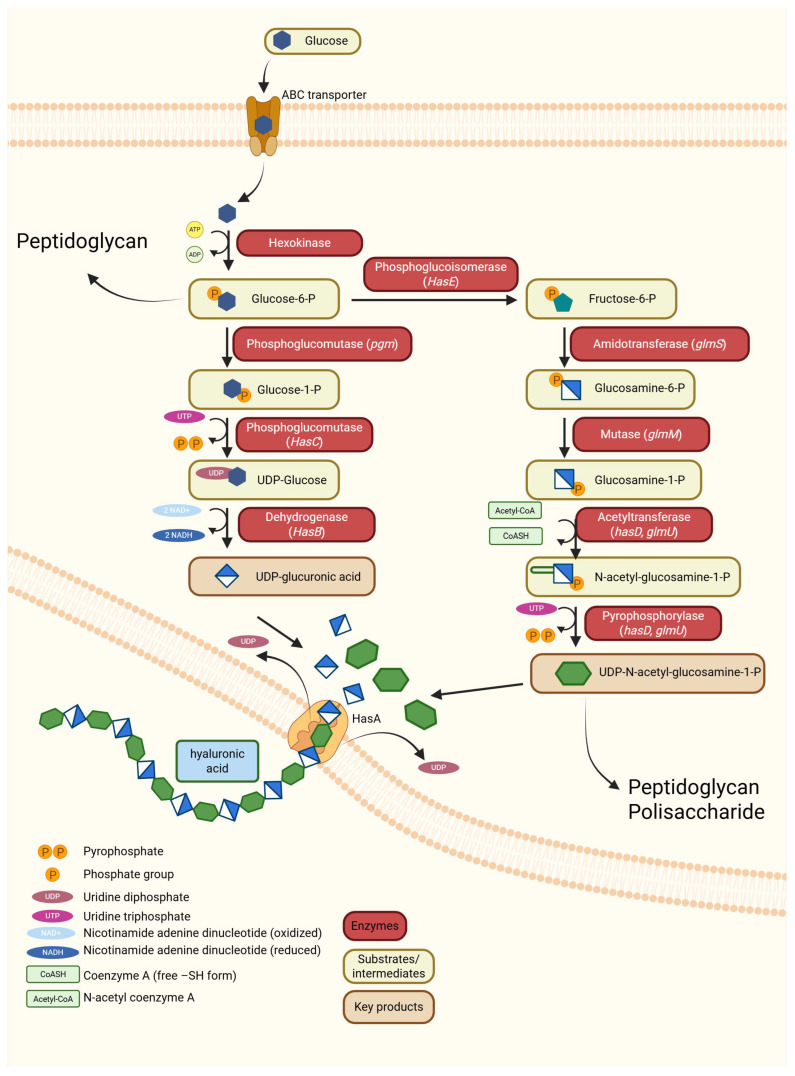
Biosynthesis pathway of hyaluronic acid in bacteria. Glucose enters the cell via an ABC transporter and is subsequently phosphorylated to glucose-6-phosphate, which is then processed through two parallel branches. In one branch of the pathway, phosphoglucomutase and *HasC/HasB* convert glucose-1-phosphate into UDP-glucuronic acid. In the second branch, fructose-6-phosphate is transformed by *GlmS*, *GlmM*, and *HasD*/*GlmU* into UDP-N-acetyl-glucosamine. These two substrates are transferred to the membrane-bound synthase HasA, which alternately polymerizes them into a linear HA chain. The polymer is secreted outside the cell [7,48]. Created by BioRender.com [51].

Bacterial HA synthesis (Figure 2, Table 1) employs fundamentally different organizational strategies. In natural producers of this compound, such as *Streptococcus zooepidemicus* HA is generated by *hasA* genes encoding Class I membrane-integrated hyaluronan synthases (HAS). These are typically located within operons containing *hasB* (UDP-glucose dehydrogenase) and *hasC* genes that provide essential precursor molecules and capsule assembly factors. This operon organization allows coordinated regulation of HA production as part of bacterial capsule formation and virulence mechanisms [8,24]. Bacterial HAS enzymes demonstrate remarkable processivity and can produce extremely high-molecular-weight HA polymers (>1000 kDa) under optimal conditions [48]. Recent mechanistic studies have shown how substrate tunnel architecture regulates substrate specificity switching and controls the alternating incorporation of sugar donors. This occurs through conformational changes that create distinct binding sites for UDP-GlcA and UDP-GlcNAc [49,50]. During polymerization step, after the sequential addition of GlcA and GlcNAc sugar residues to the growing HA chain, the polymer is extruded through the membrane directly into the extracellular space, where it contributes to the formation of the bacterial capsule.

Bacterial HA synthesis has become the foundation for industrial production, with extensive genetic engineering efforts focused on optimizing *hasA* expression, enhancing precursor supply pathways, and developing novel expression systems in heterologous hosts like *Escherichia coli* and *Bacillus subtilis*. Through membrane protein engineering and metabolic pathway reconstruction, these strategies have achieved production yields exceeding 10 g/L by coordinating UDP-sugar pool optimization and elimination of competing pathways [48].

#### 3.3.2. Hyaluronic Acid Synthesis in Fungi

##### Native Fungal Producers

Fungal HA synthesis represents the most enigmatic aspect of this comparative analysis, with native fungal HAS genes being rare and phylogenetically scattered across fungal taxa. The limited evidence for native fungal HA synthesis includes reports of HA production by certain *Cryptococcus* species [52]. However, comprehensive genomic analyses suggest that most fungi lack the enzymatic machinery for de novo HA biosynthesis, instead relying on acquisition of HA-degrading enzymes (hyaluronidases) that may contribute to nutrient acquisition, host tissue invasion, or capsule modification [52].

##### Heterologous Expression

Consequently, HA production in fungi largely relies on heterologous expression systems, in which bacterial or mammalian HAS genes are introduced into fungal hosts such as *Kluyveromyces lactis*, *Saccharomyces cerevisiae*, and *Pichia pastoris* for biotechnological applications [2]. When fungal systems are engineered for HA production, they face unique challenges related to UDP-sugar metabolism, membrane protein folding, and secretion pathway compatibility, requiring extensive metabolic engineering to establish functional HA synthesis pathways, including overexpression of UDP-glucose dehydrogenase, UDP-glucose pyrophosphorylase, and glucose-6-phosphate dehydrogenase to ensure adequate precursor supply [2,53].

## 4. Microbial Hyaluronic Acid and Hyaluronic Acid-Degrading Enzymes as Potentiators of Microbial Virulence

**Figure 3:**
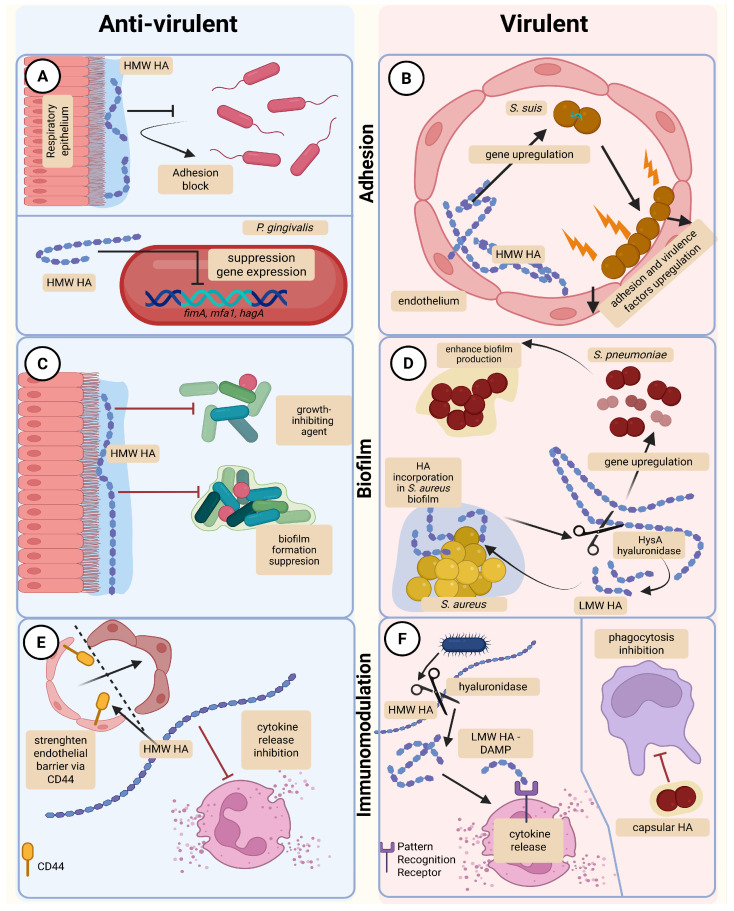
Effects of high- and low-molecular-weight hyaluronic acid on bacterial virulence and host defense. (**A**)—surface-bound HA on respiratory epithelial cells blocks bacterial adhesion, and suppresses the expression of *P. gingivalis* adhesion-related genes (*fimA*, *mfa1*, *ha-gA*). (**B**)—HMW HA enhanced the adhesion of *S. suis* to endothelial cells by increasing the expression of bacterial adhesion and virulence factors. (**C**)—HMW HA results in a suppression of biofilm formation on epithelial surfaces, whilst concurrently potentiating the effect of growth-inhibiting agents. (**D**)—LMW HA, produced by bacterial hyaluronidase (HysA), is incorporated into the *S. aureus* biofilm matrix and promotes its development. In *S. pneumoniae*, LMW HA promotes the expression of genes associated with biofilm formation. (**E**)—HMW HA stabilizes the endothelial barrier via CD44 interaction and inhibits pro-inflammatory cytokine release by immune cells. (**F**)—LMW HA functions as a DAMP, inducing the release of cytokines through pattern recognition receptors. Capsular HA protects bacteria from phagocytosis. Created by BioRender.com [55].

HA’s biodegradability, biocompatibility, and non-immunogenicity make it a unique and versatile substance in many medical and pharmaceutical applications [7,10]. Among HA’s many properties, various studies have begun to focus on HA’s ability to interfere with bacterial adhesion and biofilm formation [46,54]. Such research raises essential questions about HA’s and HAS’s potential contribution to microbial virulence, biofilm development, and as immune evasion (Figure 3).

### 4.1. Bacterial Enzymes Degrading Host Hyaluronic Acid

Since host HA plays an important role in keeping tissues hydrated and structurally supported, its degradation by bacterial enzymes may lead to reduced tissue elasticity, joint problems, and faster tissue aging. Some bacteria are able to produce hyaluronate lyase—enzyme responsible for human HA degradation. *Streptococcus dysgalactiae* subsp. *equisimilis* produces HylD, a hyaluronate lyase that exhibits significantly higher enzymatic activity than homologous enzymes at pH 6.0. These conditions are commonly found in the infected skin of elderly and diabetic patients [50]. The enzyme promotes extensive HA degradation and enhances bacterial pathogenicity through improved nutrient acquisition and tissue damage [50]. *Streptococcus pneumoniae* demonstrates variable hyaluronate lyase production, with 96% of clinical isolates showing positive enzyme activity [56]. The enzyme facilitates bacterial spread through connective tissues by degrading host HA barriers, though its role in biofilm formation appears both complex and time-dependent [57]. Likewise, *Clostridium perfringens* produces hyaluronate lyase HysA as its primary HA-degrading enzyme, distinct from other hyaluronidases, enabling the bacterium to assimilate host-derived extracellular mucosubstances and enhance invasive capacity [58]. *Streptococcus suis* serotype 2 demonstrates strain-dependent, complex interactions with host HA, the nature and extent of which are closely associated with strain virulence. Accordingly, exogenous HA modulates bacterial adhesion to brain microvascular endothelial cells, increases virulence factor expression, and enhances pro-inflammatory cytokine secretion [59]. Interestingly, the most virulent isolates (ST1 strains) lack hyaluronate lyase activity [59], suggesting that not only HA reduction but also HA accumulation during infection may contribute to pathogenesis.

However, enzymatic degradation is not the only way for HA breakdown. As we mentioned earlier (Section 3.2), HA can also be destroyed non-enzymatically by highly oxidizing ROS, including superoxide (O_2_^−^), hydrogen peroxide (H_2_O_2_), nitric oxide (NO), peroxynitrite (ONOO^−^), and hypohalous acids such as hypochlorous acid or hypobromous acid. All these compounds are generated during inflammatory responses in tissue inflammation, ischemia–reperfusion injury or sepsis [42].

Interestingly, although the micromolar ROS concentrations measured in vivo are considerably lower than the millimolar levels used in vitro, HA degradation still could occur. This is because ROS in vivo are not produced uniformly throughout the cell. Instead, they are generated within highly localized microdomains such as NADPH oxidase–rich regions of the plasma membrane, often directly adjacent to the extracellular matrix, al-lowing for their biological effectivity [60,61,62]. Highly reactive species such as hydroxyl radicals (OH·), hypochlorous acid, and ONOO^−^ react with HA at diffusion-limited rates, meaning that even short-lived, low-concentration bursts can induce HA chain scission [63,64]. Additionally, chronic oxidative exposure and synergistic activity with hyaluronidases further promote HA de-polymerization in disease states [39].

Some Gram-negative bacteria such as *Salmonella*, and certain *E. coli* strains can produce high levels of ROS, as a metabolic byproduct of their own aerobic respiration or to damage competing bacteria. For example, *Escherichia coli* exposed to various forms of attacks mediated by the type VI secretion system (T6SS), P1*vir* phage, and polymyxin B produced high levels of ROS in target cells, indicating that different forms of lethal attack enriched environment in these species [65,66]. Although there is no direct evidence linking microbial ROS to HA degradation, by analogy to mammalian ROS, microbial oxygen species could hypothetically serve as an additional source of ROS capable of degrading HA. However, further research is needed to fully confirm this possibility.

As evidenced by the examples cited, bacterial degradation of HA opens the gateway for infection and promotes dissemination into deeper tissue layers. Another aspect of this degradation is the generation of short HA fragments (LMW HA), which may contribute to the amplification of inflammatory responses. It was confirmed that, HA activates cytosolic phospholipase A_2_α and stimulates prostaglandin PGE_2_ production from arachidonic acid in human monocytes and macrophages. Next, HA acts as a danger-associated molecular pattern (DAMP) through toll-like receptor 2 (TLR2) and toll-like receptor 4 (TLR4) innate immune receptors, sustaining cytokine and chemokine release. Furthermore, it influences macrophage differentiation toward M1 phenotype which is strongly associated with a pro-inflammatory response [65,66].

Therefore, instead of merely initiating an essential defense strategy mediated by multiple mechanisms of innate immunity, prolonged LMW HA signaling can lead to detrimental outcomes, such as exacerbation of chronic inflammatory diseases (e.g., asthma, COPD, rheumatoid arthritis) [66]. Likewise, it has been shown that HA progressively accumulates in lymph nodes and splenic germinal centers during autoimmune diabetes development in murine model, and this accumulation is accompanied by a shift from high to low molecular mass HA fragments [67].

### 4.2. Bacterial Hyaluronic Acid in Biofilm Formation

The microbial biofilm forms a complex environment that equips the bacterial community with the capacity to withstand both biotic and abiotic stresses. Furthermore, it provides pathogens with powerful survival advantages such as tissue damage, antibiotic resistance, persistence, and immune evasion making infections harder to control. Research shows that bacterial biofilms are linked to nearly 80% of recurring microbial and chronic infections in humans, highlighting their major role in disease persistence and resistance to treatment [46,54].

The study by Yadav et al. (2013) investigated how HA derived from capsules of common Gram-positive bacteria affects biofilm formation and gene expression in other species [46]. They found that HA significantly supports pneumococcal growth both in planktonic (free-floating) form and within thick, organized biofilm structures, as demonstrated through crystal-violet microtiter plate assays and scanning electron microscopy. Furthermore, gene expression analysis showed that HA presence upregulated several virulence and biofilm-related genes (including *nanA*, *nanB*, *bgaA, strH*, *luxS*, *hysA*, *ugl*, and *PST-EIIA*) in planktonic cells, while downregulating *lytA* and *comA* genes. In biofilms grown with HA, genes like *luxS, hysA, ugl*, and *PST-EIIA* were upregulated by more than 2-fold. The findings suggest that HA from other streptococcal species can enhance *S. pneumoniae* biofilm development and virulence gene expression, potentially contributing to bacterial pathogenesis and persistence [46].

*Staphylococcus aureus* can utilize host-derived HA as a structural component of its biofilm matrix. These bacteria secrete hyaluronidase (HysA) to cleave HA during infection, while mutant strains deficient in this enzyme accumulate more biofilm biomass than wild-type strains. The enzyme HysA, therefore, fulfills a dual role: it facilitates biofilm dispersal when bacterial dissemination is advantageous, while its absence allows HA accumulation during biofilm establishment.

In vivo studies reveal that *hysA* mutants display impaired dissemination from biofilm-associated infection, highlighting the critical role of HA modulation in facilitating bacterial spread. Confocal microscopy reveals that HA becomes directly incorporated into the biofilm matrix, serving as a structural component [68]. Importantly, exogenous addition of purified HysA enzyme can disperse HA-containing biofilms, while a catalytically inactive enzyme has no effect [68]. Streptococcal hyaluronidases similarly degrade capsule- or host-derived hyaluronan to generate oligosaccharides that can serve as carbon sources and alter biofilm biomass, thereby promoting expansion or controlled dispersal depending on environmental cues and regulatory contexts [69,70].

Similarly to certain Gram-positive bacteria, HA can also be produced by selected pathogenic yeasts. Among them is the opportunistic pathogen Cryptococcus neoformans, the causative agent of cryptococcosis, which leads to severe meningoencephalitis in immunocompromised patients [71]. In this yeast, HA constitutes part of the polysaccharide capsule forming fibrous structures, and HA-dependent virulence has been linked to enhanced adhesion to human-brain microvascular endothelial cells [52]. The role of HA in pathogenesis of *C. neoformans* was further demonstrated in an in vivo *Caenorhabditis elegans* model, where the wild-type strain expressing hyaluronic acid synthase CPS1 was more virulent than its CPS1-deficient mutant [52].

### 4.3. Bacterial Hyaluronic Acid in Immune Evasion

Bacterial HA represents one of the most elegant immune evasion strategies employed by pathogenic bacteria. Unlike synthetic polymers, bacterial HA is structurally identical to mammalian HA, allowing pathogens to exploit the host’s tolerance to self-antigens [72]. This molecular mimicry, combined with active immunomodulatory mechanisms, enables bacteria to establish persistent infections while evading both innate and adaptive immune responses [73,74]. One of the most powerful mechanisms of innate immunity is phagocytosis which enables the engulfment of pathogens and their killing through both oxygen-dependent and oxygen-independent mechanisms. The simplest way for pathogen to avoid phagocytosis is by preventing direct recognition by phagocytic cell receptors (PRRs) which occurs prior to adherence. This purpose may be achieved by modifying PRR ligands—pathogen associated molecular patterns (PAMPs) or by masking them via production of capsules. Consequently, encapsulated bacteria are generally less efficiently phagocytosed than non-encapsulated strains as their capsule provides both a physical and immunological barrier [75].

Adherence and phagocytosis studies revealed that the encapsulated bacteria *S. equi* subsp. *zooepidemicus* containing HA-enriched capsules adhered significantly more to hu-man cervical adenocarcinoma of HeLa cells and were less phagocytosed by murine macrophages compared to non-encapsulated bacteria [76]. The impact of HA on capsule virulence was proven for the first time several decades ago by Kass and Seastone who demonstrated that when mucoid capsules of group A and C streptococci were degraded by hyaluronidases, their infectivity decreased in mice, as their susceptibility to phagocytosis by leukocytes increased [77].

Likewise, hyaluronated capsules together with surface M protein conferred resistance to opsonization by C3 complement component in group A streptococci and reduce their susceptibility to phagocytosis indicating that higher HA capsule expression correlates with lower C3 deposition and less efficient phagocytosis [78]. Finally, contribution of HA-containing capsule and M protein to virulence in a mucoid strain of the group A Streptococcus was documented also by Moses et al. [79]. The authors showed that wild-type mucoid M18 strain 282 with high content of capsular HA was resistant to phagocytic killing in whole human blood and 10% serum. In contrary, the acapsular mutant TX72 was highly susceptible to killing in 10% serum and moderately sensitive to whole blood. Furthermore, they showed that 50% lethal dose was increased 60-fold for acapsular mu-tant in mice. These results provide further evidence that the hyaluronic acid capsules confer resistance to phagocytosis and enhance group A streptococcal virulence [79].

## 5. Anti-Microbial and Anti-Virulent Activity of Hyaluronic Acid

### 5.1. The Direct Antimicrobial Activity of Hyaluronic Acid

The potential anti-virulence role of HA can be summarized by its ability to (1) inhibit microbial growth; (2) decrease the thickness and density of microbial biofilm; (3) increase antioxidant properties; (4) enhance endothelial/epithelial barrier; (5) play immunomodulatory activity in resolution of inflammation. Previous reports demonstrating the beneficial effects of HA on anti-virulence traits are summarized in Figure 3 and discussed in further detail below.

The direct antimicrobial effect of host-derived HA is primarily linked to its ability to suppress bacterial proliferation by acting as a bacteriostatic/growth-inhibiting agent, rather than directly killing bacteria. This activity is achieved by reducing bacterial adhesion to surfaces and preventing the formation of biofilm. One proposed mechanism suggests that excess HA works by saturating microbial HA-degrading lyases, enzymes facilitating the bacteria tissue penetration and spread. The absence of direct bactericidal activity of HA reflects its chemical nature and strong net negative charge. Since bacterial cell surface is also negatively charged, owing to anionic teichoic acids in Gram-positive and negatively charged lipopolysaccharides in Gram-negative bacteria, HA’s interactions with bacterial cells generate steric repulsion forces. Interestingly, these repulsive interactions enhance HA’s anti-fouling capacity, preventing bacterial attachment, accumulation, and surface colonization [80,81].

Bacteriostatic properties of exogenously applied HA have been demonstrated against *S. aureus* [24], while significant antiadhesive and antibiofilm activity has also been documented for *Moraxella catarrhalis* and *Haemophilus influenza* isolated from patients with respiratory tract infections [82]. In addition, a hyaluronan-like exopolysaccharide (EPS MO245) demonstrates significant anti-biofilm activity against *Pseudomonas aeruginosa* and *Vibrio harveyi*, achieving 20–50% biofilm inhibition without biocidal effects [82]. This inhibition occurs through physico-chemical interactions rather than antimicrobial activity, with the EPS creating a non-adsorbing layer that modifies bacterial-surface interactions [82]. Furthermore, the direct antimicrobial activity of HA was confirmed against uropathogenic bacteria. Clinical evidence shows that intravesical administration of HA combined with chondroitin sulfate (CS) in 276 women with recurrent urinary tract infections significantly reduced the risk of bacteriologically confirmed recurrences compared with standard management [83]. The high-molecular-weight hyaluronic acid (HMW HA) exerted also the growth-inhibiting properties against clinical vaginal isolates of pathogenic *Candida* strains including *C. albicans*, *C. glabrata*, *C. krusei*, *C. tropicalis.* Importantly, the fungistatic effect of HA was enhanced in combination with *Lactobacillus crispatus* lyophilised supernatant [84]. Overall, HA exhibits excellent anti-fouling properties largely attributable to its high hydrophilicity, which creates a strongly hydrated surface layer that inhibits microbial attachment and subsequent biofilm formation [85].

Reported values for HA antimicrobial and antibiofilm endpoints can vary significantly due to factors such as the experimental system, organism, HA molecular weight, and formulation. Studies that quantify these metrics demonstrate that minimum inhibitory concentration (MIC) and minimal bactericidal concentration (MBC) measured against planktonic cells, as well as minimum biofilm inhibitory concentration (MBIC) assessed against biofilm formations, are distinct and non-interchangeable endpoints and cannot be measured objectively [24,50,82,86]. In the study by Pirnazar et al., high concentrations of medium-MW HA had the greatest bacteriostatic effect, particularly against the *Actinobacillus actinomycetemcomitans*, *Prevotella oris*, *Staphylococcus aureus* and *Cutibacterium acnes* strains. All six bacterial strains were inhibited by the 1.0 mg/mL concentration of high MW HA, which had the greatest overall bacteriostatic effect [87].

In addition to anti-adhesive and anti-biofilm properties, HA can also attenuate pathogen virulence at the molecular level. HMW HA (1.5 MDa) significantly downregulates virulence-associated genes in *Porphyromonas gingivalis*, including *fimA*, *mfa1*, *hagA*, *rgpA*, and *kgp* [86]. These genes encode fimbrial proteins, adhesins, and proteases that collectively enable *P. gingivalis* to adhere to host tissues, invade cells, evade immune defenses, and mediate tissue damage. The effect is dose-dependent, with greater downregulation observed at lower concentrations (1 mg/mL) [86]. This HA-dependent modulation represents a direct antimicrobial mechanism distinct from traditional antibiotics which generally target essential cellular processes such as cell wall synthesis, protein synthesis, or DNA replication. Concentration-dependent modulatory effects of HA on bacterial virulence have also been demonstrated across multiple other species [11,88] with lower concentrations frequently producing more pronounced effects, suggesting the existence of optimal therapeutic windows for HA-based interventions.

### 5.2. The Indirect Immunomodulatory Antimicrobial Activity of Hyaluronic Acid

As discussed above, HA can directly prevent intensification of bacterial infection or contamination. However, HA is likely to have more complex roles beyond direct antimicrobial action. Equally important functions may derive from its indirect immunomodulatory effects. To date the best characterized innate immune processes modulated by HA are phagocytosis and the anti-infective inflammatory response. Both responses contribute to pathogen clearance and infection control through self-limiting reactions that minimize the risk of the development of excessive or chronic inflammation.

HA is recognized as a crucial regulator and mediator of inflammation. The role of HA in inflammation seems to be bimodal and strictly depends on the molecular size of this polymeric GAG—while HMW HA has general anti-inflammatory and immunosuppressive effects by regulating endothelial cells and promoting vascular stability, the LMW HA fragments have pro-inflammatory activities, increasing vascular permeability and promoting the recruitment of inflammatory cells [89]. HMW-HA strengthens endothelial barrier via CD44, thus reducing permeability. The protective effects of HMW-HA have been demonstrated in LPS-induced models, where it reduces pulmonary vascular hyperpermeability, induces actin cytoskeletal reorganization, and enhances endothelial barrier function [90]. Moreover, HMW HA inhibits secretion of key proinflammatory cytokines, chemokines and arachidonic acid metabolites from immune and nonimmune cells. HMW-HA also reduces the production of interleukin 1-β (IL1-β), inhibits the activation of the TLR-4 signalling pathway, and suppresses the activity of metalloproteinase 9 (MMP9) which degrades components of the extracellular matrix, and is elevated during inflammatory responses Furthermore, HA inhibits arachidonic acid release (an important source of mediators of inflammation) from human synovial fibroblasts [91]. In contrast, LMW-HA acts as an endogenous danger signal, known as a danger-associated molecular pattern (DAMP), under oxidative stress and is responsible for endothelial barrier dysfunction [92].

It is important however to add, that there are also some inconsistencies in the literature on the molecular-weight–dependent immunomodulatory roles of HA. Indeed, reports differ on whether low- or high-molecular-weight HA predominates in driving or resolving inflammation, and these discrepancies may arise from variations in HA purity, preparation methods, cell models, and experimental conditions. In line with this LMW-HA protects against intestinal inflammation upregulating intestinal tight junctions and enhancing intestinal barrier function [93]. Alternatively, HMW-HA promotes fibroblast activation in fibrosis and drives a more invasive, profibrotic fibroblast phenotype [94]. Summing up, there is still need to better define inflammatory effects of specific HA size fractions.

It has been also demonstrated that specific-sized HA fragments promote increased intracellular expression of the key innate antimicrobial peptide such as human β-defensin-2 (HBD2) in intestinal epithelial cells, both in vitro and in vivo [95]. This finding suggest that HA can actively modulate mucosal innate antimicrobial defense. This defensin is also released in response to bacterial stimulation by both respiratory and intestinal cells [88,89]. In vitro studies have shown that HBD-2 exhibited strong bactericidal activity against several clinically relevant nosocomial strains including *Acinetobacter baumannii*, *P. aeruginosa*, *Enterococcus faecalis*, *Enterococcus faecium* and *S. aureus* in vitro [96]. More recent evidence further confirmed its antibiofilm effectiveness against *P. aeruginosa* [97,98]. The role of HBD-2 in the host immune response has also been highlighted in women with intra-amniotic infection, where microbial invasion of the amniotic cavity was associated with a marked increase in amniotic fluid concentrations of HBD-2 [99].

## 6. Hyaluronic Acid in Medical Practice

Products containing HA come in many pharmaceutical forms and are registered as medicinal products, medical devices, and cosmetics. HA is used in many fields of medicine: ophthalmology, rheumatology, dental surgery, otolaryngology, gynecology, urology, orthopedics, and aesthetic medicine. In ophthalmology, it is used to treat people with dry eye syndrome. It is the main ingredient in eye drops and contact lens solutions, and is used in cataract surgery. In rheumatology and orthopedics, it is used for intra-articular injections, and in otolaryngology—to stimulate and regenerate the eardrum and vocal cords. In aesthetic medicine, it is primarily used to increase skin firmness and elasticity, improving its quality. Scientific reports from the Journal of Drugs in Dermatology have shown that 0.1% hyaluronic acid present in cosmetic formulations significantly improves the skin hydration and elasticity of the study participants [100].

LMW HA (50 and 130 kDa) is used in preparations for wrinkle reduction, which is related to its ability to penetrate into the deeper layers of the epidermis, compared to high-molecular-weight HA. HMW HA creates a hydrophilic film on the skin surface, which protects the stratum corneum from external factors and reduces trans epidermal water loss (TEWL), resulting in better hydration of the epidermis. In cosmetic formulations, hyaluronic acid acts as a humectant, maintaining moisture and preventing the preparation from drying out [101,102].

Much more lasting effects of hyaluronic acid are achieved by injecting it into the dermis using subcutaneous injections, which is why HA is used in modern aesthetic medicine as a component of fillers [100,101,102]. Hyaluronic acid-based fillers are used to reduce static wrinkles, lift and shape cheeks, correct facial features and the shape of the lips, nasolabial folds, and chin, and improve hydration and elasticity of the skin on the face, neck, décolleté, and hands [103,104].

In dermatology, preparations containing hyaluronic acid accelerate the healing of post-acne lesions, manifesting as spots, discoloration, or uneven skin tone. HA also contributes to the stabilization of tissue structure, scarring, and wound healing. Its physicochemical and biological properties render it a versatile therapeutic agent for promoting wound repair and for adjunctive antimicrobial strategies in infected or hard-to-heal wounds. HA contributes structurally to ECM hydration and viscoelasticity while engaging cell-surface receptors and modulating various biological processes, including inflammation, angiogenesis, and cell migration—functions that have been exploited in biomaterials and clinical formulations for wound care [105,106,107,108]. These biological actions underpin the therapeutic relevance of HA in wound healing applications. Preclinical and clinical studies indicate that HA-containing hydrogels, dressings, microbeads, and composite scaffolds enhance re-epithelialization, granulation tissue formation, and neovascularization while reducing excessive inflammation and scarring compared with standard treatments [109,110,111,112,113]. Currently, research is being conducted on biological dressings based on hyaluronic acid, which are claimed to heal even deep wounds within a few days without stitches and without leaving scars [114,115].

For many years, there has been an increase in the incidence of skin allergies, especially in developed countries. Many scientific studies link this situation to the increased use of synthetic substances present in cosmetic products. Exposure of the skin’s microbiota to such compounds may alter this ecosystem, as well as reducing its biodiversity. It has been shown that the use of moisturizing cosmetic products significantly reduces the number of *Cutibacterium* spp. bacteria, a genus abundant on the face, chest, and back. This relationship was particularly observed in people with oily skin. This condition is most likely related to the fact that moisturizing products reduce sebum secretion, which is a source of nutrients hydrolyzed by *Cutibacterium* spp. [103].

## 7. Potential Complications Resulting from the Use of Hyaluronic Acid

### 7.1. General Safety Profile of Hyaluronic Acid

In today’s world, where the pursuit of eternal youth and physical fitness reigns supreme, interest in treatments using tissue fillers, particularly hyaluronic acid-based preparations, continues to grow. The particular popularity of procedures using hyaluronic acid fillers may be due to the possibility of correcting them with hyaluronidase, making the treatment potentially reversible [116]. HA-based preparations are considered safe. It should be noted that this is a highly hygroscopic polymer, 1 g of which effectively retains up to 6 l of water. It is a natural substance that naturally occurs in the human body and does not cause an immune reaction. Allergy to pure hyaluronic acid is unlikely. More than half of the body’s HA reserves are found in the skin, although large amounts are also found in the vitreous body of the eye, the umbilical cord, muscles, including the heart muscle, in synovial fluid, and in the oral mucosa [117,118]. HA is also found in bacterial capsules, e.g., in the genus *Streptococcus* spp., where it protects bacterial cells from the host’s defense mechanisms, and has also enabled the possibility of industrial production of this compound and its subsequent use in cosmetology and aesthetic medicine [119].

### 7.2. Allergic Reactions and Contaminants

However, in people with a diagnosed severe allergy, even minimal amounts of allergens can cause allergy symptoms [120]. Allergy symptoms appear in response to the presence of contaminants in the preparation—such as preservatives or contaminants remaining after the HA production process, as well as residues of rooster combs or bacteria used as a source of HA acid [121,122,123,124].

### 7.3. Early and Late Procedure-Related Complications

In addition to allergies, there are other complications after using HA. These are classified as early and late complications. Early complications result from procedural or hygiene errors during the procedure or from technical errors caused by improper filler injection technique [124]. Using tissue fillers in invasive procedures carries a significant risk of bacterial infection due to the disruption of the skin barrier and the introduction of microorganisms into the tissue during injection. Recently, there has been a marked increase in interest in delayed infections occurring after tissue filler injection. This is primarily due to these infections frequently being misdiagnosed as delayed hypersensitivity reactions, resulting in inappropriate treatment [22,125].

Given that every microorganism has pathogenic potential, proper sterilization of instruments and appropriate use of cosmetic preparations play a key role in preventing complications after cosmetic procedures.

Late complications appear several weeks or months after the filler is injected into the skin. They most often take the form of nodules, some of which are purulent and inflammatory, and even occur in the form of fistulas [126,127].

### 7.4. Biofilm-Associated Infections and Their Microbiology

In recent years, increasing attention has been paid to other delayed reactions following the injection of tissue fillers. These reactions also take the form of nodules, but in this case they are infectious and are caused by the presence of a bacterial biofilm [126,128]. In one series of 61 patients with late or delayed filler-related complications, 42 cases (69%) presented nodules, 15 (25%) chronic inflammatory reactions, and 4 (7.5%) overt infection; bacterial culture in 7/61 showed organisms such as *Staphylococcus aureus*, *S. epidermidis*, *S. saprophyticus* and *Mycobacterium abscessus*. It has been suggested that tissue filler preparations promote the proliferation of microorganisms and biofilm formation, which would explain the occurrence of most complications [129,130].

Engineering HA with antimicrobial agents is a preventive strategy that can reduce colonization and biofilm formation at the biomaterial surface. Multiple recent preclinical studies demonstrate that HA matrices loaded or chemically modified with antimicrobial agents (e.g., iodine, silver nanoparticles, ZnO, CuS, metal–organic frameworks, antimicrobial peptides) confer bactericidal or anti-biofilm activity while maintaining pro-healing properties [109,110,112,131,132].

### 7.5. Diagnostic Challenges in Biofilm-Related Infections

Complications after the use of tissue fillers, such as infections caused by biofilms pose a significant diagnostic challenges. Traditional methods of identifying microorganisms using cultures on microbiological media and the collecting material from the patient using swabs from diseased areas are insufficient. Microorganisms that form biofilms are characterised by slowed cellular metabolism, meaning that the above methods may produce false negative results when isolating microorganisms [133]. It has been shown that biofilms formed in tissue fillers in cases of delayed infection are most often multispecies, with a predominance of the genus *Pseudomonas* and other microorganisms that are part of the skin’s microflora, e.g., *Streptococcus* spp. *Cutibacterium* spp., *Staphylococcus* spp., *Acinetobacter* spp. [133]. As the treatment of these infections is usually complicated and difficult, with scars or facial deformities being highly undesirable consequences, the literature indicates the need for an interview with the patient prior to treatment with a tissue filler.

### 7.6. Contraindications and Patient Selection

Contraindications include skin infections, dental abscesses, generalized infections, autoimmune conditions, recent antibiotic therapy, rosacea, seborrheic dermatitis, and transplants. Skin diseases associated with skin microbiome disorders, such as atopic dermatitis or acne, are an absolute contraindication to tissue filler injections. In addition to interviewing the potential patient, it is important to emphasize the importance of preventive measures such as disinfecting the skin before introducing the filler into the tissues. Furthermore, it has been shown that repeatedly piercing the skin barrier with a needle can lead to infection [126,127,134].

### 7.7. Management of Suspected Infectious or Biofilm-Related Complications

In circumstances where infection or a biofilm-associated complication is suspected, management differs from that of a simple allergic type-reaction protocol. First line approaches generally comprise systemic antibiotics, which are directed towards the skin flora, such as *Staphylococcus* and *Streptococcus* species. In the context of HA fillers, the application of hyaluronidase, an enzyme that facilitates degradation, has been proposed as a method for the removal of the substrate that supports the development of a biofilm. In cases of persistent nodules or granulomas, surgical excision or drainage is indicated when more conservative management has failed to achieve the desired outcome [135,136,137,138].

### 7.8. Prevention Strategies

This highlights the need to maintain hygiene and the highest possible level of cleanliness during the procedure, but the prevention of delayed infections caused by biofilm does not depend solely on the person performing the filler injection. It has been shown that before undergoing this type of treatment, one should refrain from using makeup for up to a month beforehand [133]. An important factor predisposing to delayed infections after tissue filler injections is repeated procedures, as the needle piercing the biofilm formed in the previously administered filler activates it and results in visible symptoms, such as abscess formation. The long time before the onset of infection symptoms is explained by the dormancy of microorganisms in the biofilm, which, as a result of a decrease in the host’s immunity or mechanical action on the biofilm, become active and cause an active infection.

In order to prevent the formation of biofilm in the filler and tissues, the potential for prophylactic use of antibiotics during the procedures has been emphasized, which at present seems to be the most effective protection against undesirable complications in the form of infection [129]. Unfortunately, the increasing resistance to antibiotics makes the use of this type of procedures questionable and unwise.

Maintaining the highest possible hygiene during procedures is essential. Additional prevention measures include avoiding makeup for up to a month before the procedure [133].

Repeated filler procedures increase risk, as needle puncture of an existing biofilm may reactivate dormant microorganisms, causing delayed infection.

Prophylactic antibiotics have been suggested as a preventive measure, although rising antimicrobial resistance makes this approach controversial [129].

### 7.9. Novel HA Conjugates and Advanced Biomaterials

Despite the widespread use of hyaluronic acid in medicine and its dualistic nature, new research is currently being conducted on innovative combinations of HA with other substances [108,113,139,140]. HA conjugates with peptides, growth factors, or antimicrobials represent a recently discovered class of targeted therapeutic agents that combine the biocompatible, extracellular matrix (ECM)-mimetic scaffold properties of HA with ligand-driven targeting, controlled release, and multimodal bioactivity. The high affinity of HAfor CD44 and other cell-surface receptors facilitates active targeting of HA-functionalized carriers (e.g., liposomes, nanoparticles, hydrogels) to cells that overexpress these receptors. This process enhances local payload delivery and therapeutic index [139,140].

The conjugation of bioactive peptides to HA scaffolds or HA-coated nanomaterials allows for the presentation of adhesive, antimicrobial, or signalling motifs in a matrix-bound fashion [141]. The result is enhanced cell adhesion, in addition to the direction of differentiation. Furthermore, it has been demonstrated to exert anti-biofilm activity, whilst preserving the integrity of the scaffold. Chemically binding growth factors (e.g., EGF, VEGF) or incorporating them into HA hydrogels stabilizes labile proteins, enables sustained release, and maintains bioactivity in situ to promote angiogenesis and tissue regeneration, as demonstrated by HA hydrogels that release EGF and VEGF to accelerate skin and bone repair in preclinical models. However, the number of large randomised clinical trials remains limited, and ongoing improvement of conjugation chemistries, release kinetics and safety profiles is necessary for the trials to achieve broader clinical adoption [142,143,144].

## 8. Conclusions

Hyaluronic acid exhibits a dualistic role in microbial interactions, functioning both as an antimicrobial agent and as a virulence-enhancing factor depending on its molecular weight, structural configuration, and biological context. Understanding these contrasting activities is essential for developing HA-based therapeutics that maximize antimicrobial benefits while minimizing the risk of enhancing pathogenicity.

Growing interest in hyaluronic acid has expanded beyond its native biological functions toward engineering HA-based systems with enhanced antimicrobial or immunomodulatory performance. One promising direction involves the development of HA conjugates with antimicrobial peptides, where HA serves as a biocompatible carrier enabling sustained release, reduced peptide degradation, and the improved targeting of bacterial biofilms. Similarly, HA–antibiotic or HA–metal nanoparticle conjugates are emerging as strategies to enhance drug penetration into biofilms and reduce off-target cytotoxicity.

Another rapidly evolving field concerns HA conjugation with growth factors or cytokines to promote controlled tissue regeneration alongside antimicrobial activity—an approach particularly relevant for chronic wounds. Incorporating HA into “smart” hydrogels or stimuli-responsive scaffolds further allows for localized, on-demand release of therapeutic molecules in response to pH, enzymatic activity, or bacterial load.

Overall, exploring these advanced HA-based hybrid systems may provide powerful tools for targeted therapy, improved biofilm management, and combined antimicrobial–regenerative interventions. Future research should therefore prioritize the controlled manipulation of HA properties to harness its protective effects without unintentionally promoting microbial virulence.

## Figures and Tables

**Table 1:** Characteristics of HAS proteins among vertebrates and bacteria.

Protein	Key Characteristics	Class	References
**Human HAS1**	Membrane-integrated bifunctional synthase Contains GT-2 catalytic module with multiple transmembrane segments Low basal activity, upregulated by inflammatory signals Alternately transfers UDP-GlcA and UDP-GlcNAc	Class I membrane-integrated Hyaluronan synthase	DeAngelis & Zimmer (2023). [7]
**Human HAS2**	Principal mammalian isoform for high-MW HA synthesis Essential for development (embryonic lethal when deleted) Tightly regulated, major contributor to tissue HMW-HA	Class I membrane-integrated GT-2 Hyaluronan synthase	DeAngelis & Zimmer (2023). [7]
**Human HAS3**	Produces lower-molecular-weight HA chains Preferentially yields smaller HA polymers Cell cycle regulated expression	Class I membrane-integrated Hyaluronan synthase	DeAngelis & Zimmer (2023). [7]
***S. pyogenes*** **HasA**	Single membrane-embedded GT-2 module Part of has operon (hasA/hasB/hasC) Essential for virulence and immune evasion	Class I membrane-integrated GT-2 Hyaluronan synthase Reducing-end addition mechanism	Weigel (2015). [25] Wessels (2019). [26]
***S. zooepidemicus* HasA**	Membrane-integrated GT-2 synthase High HA production capacity Used extensively for industrial HA production Biotechnology applications due to high yield	Class I membrane-integrated GT-2 Hyaluronan synthase	de Oliveira et al. (2016). [2] Blackburn et al. (2018). [27]
***P. multocida*** **PmHAS**	Catalyzes HA capsule polymerization	Class II dual-domain GT-2 Two separate catalytic modules Different from Class I mechanism	Pasomboon et al. (2021). [28] van der Vlist & Loos (2010). [29]

## Data Availability

No new data were created or analyzed in this study. Data sharing is not applicable to this article.

## Abbreviations

The following abbreviations are used in this manuscript:

HA	hyaluronic acid, hyaluronan
GAGs	glycosaminoglycans
CaMKKβ	calcium-dependent kinases
AMPKα	AMP-activated protein kinase
ULK1	autophagy regulatory proteins
RHAMM	receptor for HA-mediated motility
LYVE-1	lymphatic vessel endothelial HA receptor 1
TSG-6	TNF-stimulated gene 6
ECM	extracellular matrix
HasA, HAS	hyaluronan synthase
HYAL	hyaluronidase
ROS	reactive oxygen species
T6SS	type VI secretion system
LMW HA	low-molecular-weight hyaluronic acid
DAMP	danger-associated molecular pattern
HysA	hyaluronidase
PRRs	phagocytic cell receptors
PAMPs	pathogen associated molecular patterns
CS	chondroitin sulfate
HMW HA	high-molecular-weight hyaluronic acid
TLR2	toll-like receptor 2
TLR4	toll-like receptor 4
MMP9	metalloproteinase 9
HBD2	human β-defensin-2
TEWL	trans epidermal water loss
